# High Plasticity of the Gut Microbiome and Muscle Metabolome of Chinese Mitten Crab (*Eriocheir sinensis*) in Diverse Environments

**DOI:** 10.4014/jmb.2011.11018

**Published:** 2020-11-16

**Authors:** Xiaowen Chen, Haihong Chen, Qinghua Liu, Kangda Ni, Rui Ding, Jun Wang, Chenghui Wang

**Affiliations:** 1 School of Medicine, Tongji University, 239 Siping Road, Shanghai 200433, P.R. China; 2 Key Laboratory of Freshwater Aquatic Genetic Resources, Ministry of Agriculture/National Demonstration Center for Experimental Fisheries Science Education/Shanghai Engineering Research Center of Aquaculture, Shanghai Ocean University, Shanghai 01306, P.R. China; 3 Fusuile Biotechnology Co., Ltd., No. 1999, Beixing Road, Shanghai 202179, P.R. China

**Keywords:** Gut microbiome, metabolome, *Eriocheir sinensis*, phenotypic plasticity

## Abstract

Phenotypic plasticity is a rapid response mechanism that enables organisms to acclimate and survive in changing environments. The Chinese mitten crab (*Eriocheir sinensis*) survives and thrives in different and even introduced habitats, thereby indicating its high phenotypic plasticity. However, the underpinnings of the high plasticity of *E. sinensis* have not been comprehensively investigated. In this study, we conducted an integrated gut microbiome and muscle metabolome analysis on *E. sinensis* collected from three different environments, namely, an artificial pond, Yangcheng Lake, and Yangtze River, to uncover the mechanism of its high phenotypic plasticity. Our study presents three divergent gut microbiotas and muscle metabolic profiles that corresponded to the three environments. The composition and diversity of the core gut microbiota (Proteobacteria, Bacteroidetes, Tenericutes, and Firmicutes) varied among the different environments while the metabolites associated with amino acids, fatty acids, and terpene compounds displayed significantly different concentration levels. The results revealed that the gut microbiome community and muscle metabolome were significantly affected by the habitat environments. Our findings indicate the high phenotypic plasticity in terms of gut microbiome and muscle metabolome of *E. sinensis* when it faces environmental changes, which would also facilitate its acclimation and adaptation to diverse and even introduced environments.

## Introduction

Acclimation is the premise for survival when species shift to a different environment. This shift imposes strong selective pressures on species, which will shape their phenotype rapidly and dramatically [1, 2]. Numerous studies have emphasized that the ability of organisms to acclimate to new habitat conditions depends on their phenotypic plasticity attributes, which are a rapid-response mechanism that enables organisms to survive in changing environments [3]. Natural biological phenomena such as migration, biological invasion, and domestication cause environmental changes and lead to phenotype variations, physiological changes, and genomic adaptation in nature [4, 5]. When environments change rapidly, adaptive phenotypic plasticity ameliorates the negative effects of environmental change on survival and reproduction [6]. Species with high phenotypic plasticity produce different phenotypes in response to rapid environmental change and eventually adapt to their new environment. Species that fail to acclimate to the changing environment are eliminated by nature [3, 7]. The phenotype variation and underlying mechanism of vertebrates, which serve as their response to environmental changes, have been extensively studied. In comparison, related studies on the phenotype plasticity of aquatic crustacean species, which are a large invertebrate group that exists and adapts well in diverse aquatic environments, are limited [1, 2].

Previous studies have indicated that the differential gene expression, epigenetic regulation, and regulated gene signaling network are the main mechanism for phenotypic plasticity [1]. In addition to the gene regulation system, recent research reported that gut microbiome communities influence host biology more than what was presumed and are a key factor that defines the hosts’ phenotypes [2, 8]. For example, gut microorganisms affect various aspects of the host, such as metabolism, growth, development, immunology, nutrition, and behavior [1,2,4,9-11]. The change of environmental factors such as temperature, pH, dietary resources, water chemistry, and salinity causes fast and profound variations in the gut metagenome of organisms [2, 12-16]. The plasticity of the gut microbiota is an essential factor that determines the phenotypic plasticity of vertebrates. It is a factor that plays a pivotal role when vertebrates acclimate and adapt to rapid environmental variations [1]. Metabolomic profiles quantify the complete set of metabolites in a cell, tissue, or organ of a species and can be applied for the assessment of the physiological condition of an organism in response to diverse genetic and environmental factors [17, 18]. Meanwhile, metabolomics is presumed to be the perfect representation of phenotype response in divergent environments [19].

The Chinese mitten crab (*Eriocheir sinensis*), which belongs to the subphylum Crustacea, is a catadromous species that lives and grows in freshwater but reproduces in a natural seawater environment [20]. *E. sinensis* inhabits diverse river systems (*e.g.*, Yangtze River, Yellow River, and Liao River basins) and inland freshwater lake systems (*e.g.*, Yangcheng, Tai, and Gucheng Lakes) [21]. Given the large consumer market, *E. sinensis* is also widely cultured in artificial ponds to provide delicacies to Chinese consumers [21]. *E. sinensis* is also cultured and lives in reservoirs, rice fields, and other associated water bodies [20, 22]. Moreover, *E. sinensis* is a notorious invasive species distributed in North America and in almost the entirety of Europe [23-25]. This species can survive and become sexually mature in diverse environments, and thus exhibits high phenotype plasticity under diverse environments. However, the underpinnings of the high phenotypic plasticity of *E. sinensis* in different environments have not yet been comprehensively explored. In this study, we conducted a gut microbiome and muscle metabolome analysis on *E. sinensis* collected from three diverse environments to uncover the response of gut microbial communities and muscle metabolome toward environmental changes and reveal the fundamental basis for the acclimation of *E. sinensis* in diverse environments.

## Materials and Methods

### Sample Collection and Ethics Statement

**Fig. 1:**
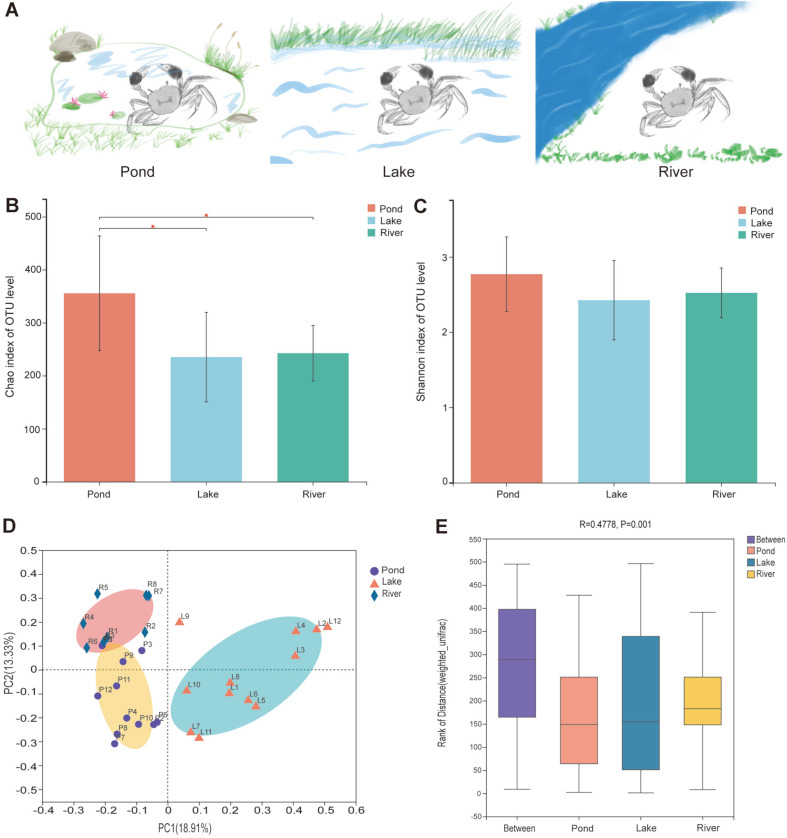
Alpha and beta diversity analyses of the gut microbiota in the three environmental groups. (**A**) Three diverse environments that *E. sinensis* inhabits; (**B**) Alpha diversity (Chao index) estimate at the OTU level in the three environmental groups, * indicate *p* <0.05 for statistical analysis; (**C**) Alpha diversity (Shannon index) estimate at the OTU level in the three environmental groups; (**D**) Beta diversity (PCoA) estimates for the bacterial communities in the three environmental groups; and (**E**) Beta diversity (ANOSIM) estimate in the three environmental groups.

Adult *E. sinensis* individuals were collected from three environments, namely, the aquaculture pond of Fusuile Biotechnology Co., Ltd. (pond group; China), a net cage in Yangcheng Lake (lake group; China), and the Yangtze River (river group; Taizhou, China) (Fig. 1A). The collected *E. sinensis* samples were immediately dissected after removal from a phosphate buffer wash, and the gut and muscle tissue from each group were sampled, snap-frozen in liquid nitrogen, and stored at −80°C in a refrigerator. Twelve biological replicates from the pond and lake groups and eight from the river group were sampled. This study was approved by the Institutional Animal Care and Use Committee of Shanghai Ocean University (Shanghai, China). The sampling procedures complied with the guidelines of the Institutional Animal Care and Use Committee on the care and use of animals for scientific purposes.

### Gut Microbiota Sequencing and Data Analysis

Gut DNA was extracted from 32 collected samples by using a DNA extraction kit (OMEGA, China) in accordance with the manufacturer’s protocols. The extracted DNA concentration and purity were evaluated by using the NanoDrop 2000 platform (China). The V3–V4 region of 16S RNA was amplified by using 338F (5′–ACTCCTACGGGAGGCAGCAG–3′) and 806R (5′–GGACTACHVGGGTWTCTAAT–3′) primers with ~468 bp PCR product. Paired-end sequencing libraries (PE300) were constructed and sequenced on an Illumina MiSeq platform (Illumina, USA).

After sequencing, raw sequencing reads were quality filtered using fastp v0.19.6 software before the analysis [26]. Subsequently, paired-end reads were merged into a consensus sequence in accordance with the information between paired-end reads with overlaps longer than 10 bp by using FLASH v1.2.11 [27]. Operational taxonomic unit (OTU) clustering was conducted using UPARSE (7.0.1090) with ≥ 97% similarity [28]. The number of OTUs was summarized with USEARCH 7.0 and an OTU data table was generated for each group [28]. The Ribosomal Database Project classifier (RDP) was used for the OTU annotation, and the representative sequences of each OTU was selected to annotate the taxonomic information with an identity threshold of 0.7 [29]. The alpha diversity of Sobs, Chao, and Shannon indices was calculated using the Mothur 1.30.2 software at OTU level [30]. The Bray-Curtis distance matrices, which were used to calculate the beta diversity, were visualized via principal coordinate analysis (PCoA). Analysis of similarities (ANOSIM) was conducted to detect the between-groups differences by using the Bray-Curtis distance implemented in QIIME software [31]. Permutational Multivariate Analysis of Variance (PERMANOVA) was conducted through the weighted UniFrac distance implemented in QIIME software [31]. Clustering of gut microbial taxa into different types was performed using the partitioning around medoids (PAM) clustering method with Jensen-Shannon divergence (JSD) and was visualized by PCoA. PICRUSt2 was applied to predict the functions of an OTU against a database of 16S bacterial sequences [32].

### Muscle Liquid Chromatography-Mass Spectrometry (LC-MS) Metabolomics Processing and Data Analysis

A total of 32 muscle samples were subjected to LC-MS for untargeted metabolomics analysis. First, 50 mg muscle tissue was added into 400 μl biochemical solution (methanol: acetonitrile = 1:1) in a clean tube and was homogenized with a high-throughput tissue crusher. Then, each sample was ultrasonically extracted and incubated at −20°C for 30 min and centrifuged at 13,000 ×g for 15 min at 4°C. Finally, the supernatant was extracted and dried. A total of 180 μl of 50% acetonitrile solution was used to redissolve the dried samples for LC-MS analysis. The LC-MS experiment was conducted with a UHPLC Q-Exactive HF-X platform (Thermo, USA). In order to assess the reproducibility and reliability of the LC-MS system, quality control (QC) samples of equal volumes from each sample were mixed and detected.

The generated raw data were processed using Progenesis QI (Waters Corporation, USA) for peak picking, peak alignment, and peak filtering. Then, the data matrices for retention time, M/Z, and peak intensity were normalized under the following conditions: 1) only the metabolites present in >80% of samples were retained; 2) the missing values were replaced with half of the minimum value; 3) the peak intensities were normalized to the total spectral intensity; and 4) log10 transformation was applied to the raw data to obtain the final data set. The normalized data were matched with the HMDB (http://www.hmdb.ca/) and Metlin (https://metlin.scripps.edu) public databases to obtain accurate qualitative results for each metabolite [33].

Positive and negative data were imported into the ROPLS v1.6.2 software package [34]. Partial least squares discriminant analysis (PLS-DA) was conducted to visualize the metabolic alterations among the three groups after mean centering and unit variance scaling. Variable importance in the projection (VIP) was adopted to rank the overall contribution of each variable to the PLS-DA model; the variables with VIP > 1.0 were considered relevant for group discrimination. Metabolites with VIP > 1 and *p* < 0.05 were considered as differential metabolites between groups and subjected to KEGG enrichment analysis by using software implemented in the Majorbio I-Sanger Cloud platform. The Spearman’s correlation coefficients between the abundance of metabolite and gut microbiome communities at the phylum level were calculated and clustered on the basis of the Euclidean distance.

## Results

### Sequencing, Richness, and Diversity Estimates of Microbiomes under Different Environments

A total of 1,433,338 sequences were obtained after sequencing the 32 samples from the three environments, and 850 different OTUs representing 359 genera were identified. The Shannon rarefaction curve for the number of reads and Shannon index at OTU level revealed the tendency of each group to exhibit plateau saturation (Fig. S1).The alpha diversity of the Chao index indicated the significant differences in community richness between pond and lake groups and between pond and river groups (*p* < 0.05; Fig. 1B). By contrast, the alpha diversity of the Shannon index indicated no significant differences in community diversity among the groups (Fig. 1C). The beta diversity analysis through PCoA indicated a significant divergence among the three groups (Fig. 1D). ANOSIM test identified large differences among the three groups (R = 0.4778 and *p* = 0.001; Fig. 1E). Meanwhile, highly divergent gut communities were observed in the PERMANOVA test (1000 permutations) by using the weighted UniFrac distance among the three environmental groups (F = 5.718, R^2^ = 0.2828, *p* = 0.001).

### Comparison of Gut Microbiome Compositions and Diversity under Different Environments

**Fig. 2:**
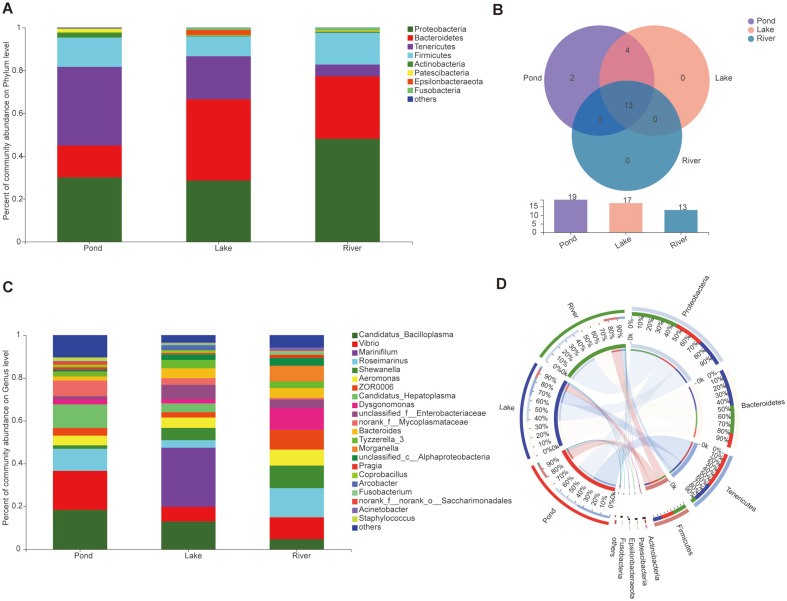
Composition and abundance of the gut microbiome communities in the three environmental groups. (**A**) Compositions and abundances of the microbiome communities at phylum level in the three environmental groups; (**B**) Venn diagram of the composition of the gut microbiome in the three environmental groups; (**C**) Compositions and abundances of the microbiome communities at genus level in the three environmental groups; and (**D**) Circos plot of the proportion of gut microbiome in the three environmental groups at phylum level.

**Fig. 3:**
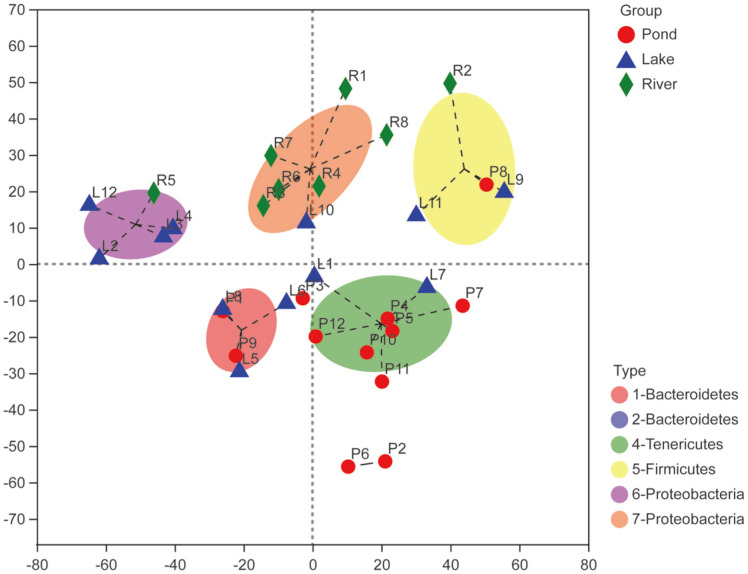
Clustering of gut microbial taxa into different types on phylum level in the three environmental groups. Note: The type with only one representing sample is not labeled in the figure.

Although Proteobacteria, Bacteroidetes, Tenericutes, and Firmicutes were dominant bacteria phyla in the three environmental groups, a divergent gut microbiome was observed among the three groups. Tenericutes (36.72%), Bacteroidetes (37.94%), and Proteobacteria (48.07%) were the most abundant bacterial phyla in the pond, lake, and river groups, respectively (Fig. 2A). WS2 and Rokubacteria were only present in the pond group. Gemmatimonadetes, Verrucomicrobia, Chlamydiae, and Planctomycetes were not identified in the river group, but these phyla were observed in the pond and lake groups (Fig. 2B). At the genus level, the three groups showed diverse bacterial community composition. *Candidatus Bacilloplasma* (18.39%) and *Vibrio* (18.30%) were the most abundant genera in the pond group; *Marinifilum* (27.60%), which only exist in the lake group, was the most dominant genus in this group; and *Roseimarinus* (13.56%) was the dominant genus in the river group (Fig. 2C). The circos plot displayed the proportion of core bacteria communities among the three environmental groups, as well as the diverse composition and diversity of the bacterial communities at phylum and genus level (Figs. 2D and S2). Seven gut microbial cluster types were identified among the three groups that represented the three different environments at the phylum level. The river group belongs to type 7-Proteobacteria, the pond group mainly belongs to type 4-Tenericutes, and the lake group mainly belongs to type 6-Proteobacteria and type 1-Bacteroidetes (Fig. 3).

**Fig. 4:**
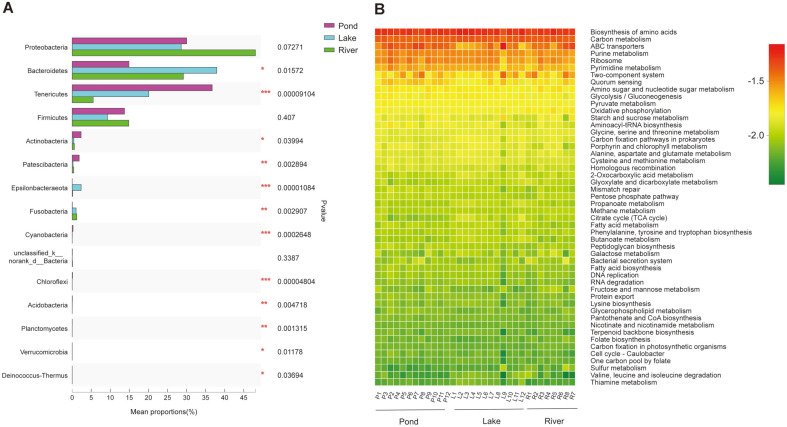
(**A**) Comparison and (**B**) functional prediction of the gut microbiome communities of the three environmental groups.

Among the three environmental groups, the relative abundances of Proteobacteria and Bacteroidetes were the highest in the river and lake groups, respectively (*p* < 0.05; Fig. 4A). Meanwhile, the abundances of Tenericutes, Actinobacteria, and Patescibacteria were highest in the pond group (*p* < 0.05; Fig. 4A). Although the three groups presented diverse gut microbiome composition and abundance, the function annotation analysis highlighted that essential pathways were shared among them. This result indicated the relative normal function of the gut in the three environments (Fig. 4B).

### Comparison of the Muscle Metabolomic Profiles under Different Environments

**Fig. 5:**
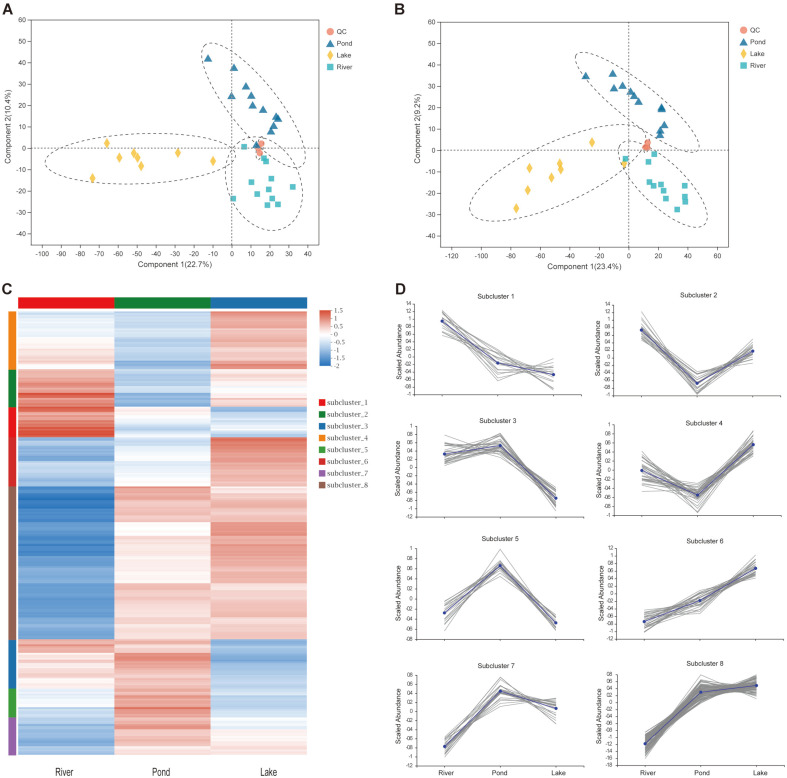
Overview and comparison of the muscle metabolomic profiles in the three environmental groups. (**A**) Score plots of PLS-DA analysis in positive mode using identified metabolites data; (**B**) Score plots of PLS-DA analysis in negative mode using identified metabolites data; (**C**) Heatmap cluster analysis for identified differential metabolites; and (**D**) Abundance pattern of identified metabolites in the eight clusters.

A total of 631 annotated metabolites (250 in positive-ionized mode and 381 in negative-ionized mode) were identified in the three environmental groups. These metabolites were classified into nine KEGG compound groups, with lipids (32.05%) and peptides (19.23%) as the top two compounds (Fig. S3). In addition, 36 KEGG pathways were annotated, and the lipid and amino acid metabolisms were the pathways with the largest annotated metabolites (Fig. S4). The score plots of the PLS-DA were generated to present a global overview of the metabolites among the three environmental groups. Both positive and negative data revealed significant discrimination among the three environmental groups (Figs. 5A and 5B).

A total of 144 differential metabolites were identified, among which 110 were upregulated and 34 were downregulated in the pond group compared with the river group. The upregulated metabolites in the pond group were enriched in riboflavin (vitamin B2) and galactose metabolism pathways (Fig. S5), whereas those in the river group were enriched in sphingolipid signaling, neurotrophin signaling pathways, arginine biosynthesis, and insect hormone biosynthesis (Fig. S5). For the lake group, 146 differential metabolites were identified, among which 123 were upregulated and 23 were downregulated compared with the river group. On the one hand, the upregulated metabolites in the lake group were enriched in taste transduction, mTOR signaling pathway, galactose metabolism, PI3K-Akt signaling pathway, and FoxO signaling pathway (Fig. S6). On the other hand, the upregulated metabolites in the river group were enriched in GABAergic synapse, choline metabolism in cancer, oxidative phosphorylation, citrate cycle (TCA cycle), and arginine biosynthesis (Fig. S6). A total of 132 metabolites were identified in the pond group, among which 54 were upregulated and 78 were downregulated compared with the lake group. The upregulated metabolites in the pond group were enriched in D-glutamine and D-glutamate metabolisms, alanine, aspartate, and glutamate metabolism (Fig. S7), whereas those in the lake group were enriched in linoleic acid metabolism, glycerophospholipid metabolism, sphingolipid metabolism, mTOR signaling pathway, and PI3K-Akt signaling pathway (Fig. S7).

**Fig. 6:**
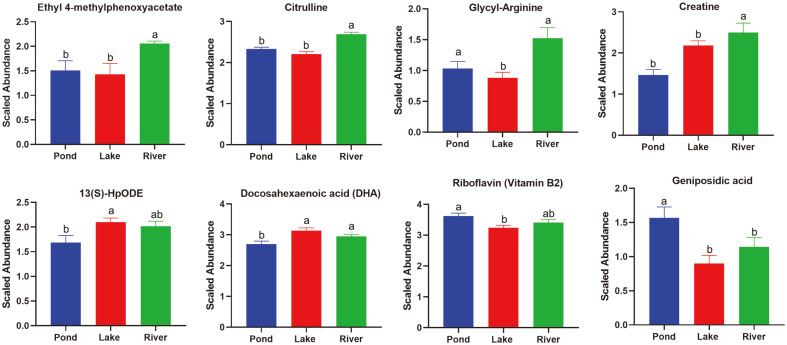
Relative abundance of the identified differential metabolites among the three environmental groups.

A total of 261 differential metabolites were identified among the three groups. The heatmap cluster analysis showed three diverse muscle metabolomic profiles among the groups on the basis of the differential metabolites (Fig. 5C). Eight clusters were clearly defined. In clusters 1 and 2, the relative abundances of sterebin E, ouabain, sphingomyelin, glycyl-arginine, citrulline, ethyl 4-methylphenoxyacetate, creatine, crocin 4, ganoderic acid H, cyanidin 3-O-dimalonyl-laminaribioside, and ganoderic acid F were the highest in the river group (Figs. 5D and 6 and Table S1). In clusters 5 and 7, the relative abundances of riboflavin, austalide H, lumichrome, nobiletin, geniposidic acid, acetylsoyasaponin A2, and tricrocin were the highest in the pond group (Figs. 5D and 6 and Table S1). In clusters 4 and 6, the relative abundances of isoleucylproline, isoleucyl-isoleucine, valyl-proline, hydroxyprolyl-lysine, docosahexaenoic acid (DHA), 15(S)-hydroxyeicosatrienoic acid, and 13(S)-hydroperoxyoctadecadienoic acid (HpODE), were the highest in the lake group (Figs. 5D and 6).

The correlation analysis between the abundance of differential metabolites and gut microbiome communities implied that in clusters 1 and 2, citrulline was positively correlated with Proteobacteria, whereas creatine, crocin 4, and ganoderic acid H were positively correlated with Bacteroidetes (Fig. S8). In clusters 4 and 6, riboflavin and geniposidic acid were positively correlated with Cyanobacteria; austalide H was positively correlated with Chloroflexi; and lumichrome, acetylsoyasaponin A2, and tricrocin were positively correlated with Tenericutes. In clusters 3 and 5, valyl-proline, hydroxyprolyl-lysine, and DHA were positively correlated with Bacteroidetes, whereas 15(S)-hydroxyeicosatrienoic acid and 13(S)-HpODE were positively correlated with Epsilonbacteraeota (Fig. S8).

## Discussion

As previously mentioned, the ability of organisms to acclimate or adapt to new habitat conditions depends on their phenotypic plasticity [1]. Given its high phenotypic plasticity, *E. sinensis* can survive and thrive in diverse water environments [21]. We identified three diverse gut microbiotas and muscle metabolomic profiles corresponding to the pond, lake, and river environments, which strongly indicated the high plasticity of the gut microbiome and muscle metabolome of *E. sinensis* in different environments.

 Numerous studies have suggested that gut microbiota could be quickly and deeply altered by changes in habitat and affect the growth and development of the hosts [1, 35, 36]. The gut microbiota inhabits the host intestine and forms a relatively stable intestinal ecological environment to acclimate to diverse environments [37]. In this study, three diverse gut microbiotas were presented with significantly different composition and diversity of core gut microbiome communities (Figs. 1 and 2). Numerous biotic and abiotic variables which are different in the three habitat types considered in this study could potentially influence the gut microbiota. The significant differences of abiotic variables are water chemistry, temperature, pH, living space, and the biotic features are prey resources and abundance, and human activity [37]. Natural animal-type food resources, such as small fishes and crustaceans, are the main prey of *E. sinensis* in the Yangtze River. Therefore, *E. sinensis* inhabiting the Yangtze River need to catch and fight for limited animal-type food resources [20]. Proteobacteria were associated with a carnivorous diet and the abundance of Proteobacteria was significantly higher in carnivorous fishes than in herbivorous ones. The large abundance of Proteobacteria might be an indication of a mainly carnivorous diet habit for *E. sinensis* in the Yangtze River [38]. Our previous study indicated that *E. sinensis* fed with only freshwater snails during aquaculture presented a high proportion of Proteobacteria, which is consistent with the results of the present work. Moreover, the high proportion of Proteobacteria might be also be associated with potential dysbiosis and disease, for Proteobacteria was considered as maleficent bacteria that cause infection [9, 39]. Regarding the lake group, in addition to the natural animal-type food resources, water plants are also abundant in Yangcheng Lake [40]. Yangcheng Lake is a suitable and famous water source for producing high-quality *E. sinensis* [41]. Bacteroidetes play important roles in lignocellulose degradation and the abundance of Bacteroidetes was highest in the lake group. This finding indicated that the gut microbiome was shaped into an efficient microbiome community for plant digestion [42]. Meanwhile, the dominant gut microbiome exhibited a relatively even distribution in the lake group compared with the river and pond ones (Bacteroidetes = 37.94%, Proteobacteria = 28.59%, and Tenericutes = 20.08%). This result signified a relatively balanced gut microbiome community in the Yangcheng Lake environment. Another type of gut microbiome was present in the pond group. Although animal-type and plant-type food resources were provided to the pond group by fish farmers to imitate the lake group environment, the environmental factors between the two environments were different and the living space in the former was smaller than that in the latter. In addition, the water quality was difficult to maintain due to the small and closed water body in the pond. Furthermore, due to the harvest season, many small wild fishes and crustaceans were provided to maintain the final weight and condition factor of adult *E. sinensis*. As a result, the water body became highly eutrophic [20]. The special artificial pond environment might cause the rapid changes of the gut microbiome in the pond group, such as the high abundance of Tenericutes and *Vibrio* in the pond group, which were associated with the eutrophic water body [43]. The gut microbiome of the three environmental groups presented significant divergence, and thus reflected their acclimation to the environment. The gut microbiome of *E. sinensis* could be easily shaped by environmental factors such as diet resources and abundances or water chemistry in the habitats.

Many studies have reported dramatic nutritional value and flavor variations in the physiological characteristics of *E. sinensis* cultured in different environments [44-46]. In addition to the gut microbiome, the muscle metabolomic profiles exhibited changes in the three environments in this study (Figs. 5 and 6). The metabolomics profiles revealed a clear separation and discrimination among the three groups which was consistent with the analysis of gut microbiome (Fig. 5). In the river group, the highest abundance was demonstrated by related metabolites, such as citrulline and glycyl-arginine in arginine biosynthesis pathway (Fig. 6). Arginine is an essential amino acid that plays important roles in increasing vasodilation, elevating blood flow to the exercising muscle, and enhancing the metabolic response to intense exercise [47]. The water flow rate in the Yangtze River is greater than those in the pond and Yangcheng Lake [48]. However, diet resources in the former were not as abundant as those in the pond and lake groups. Therefore, the *E. sinensis* in the river group required intense exercise to overcome the high water flow and compete for the limited food [20, 48]. *E. sinensis* is a catadromous species that migrate to seawater for reproduction after they get sexually mature in freshwater [49]. The high abundance of arginine would benefit the above biological process and reflect the full acclimation of the *E. sinensis* to the river environment [20]. Creatine, which is an amino acid derivative that helps increase muscle strength and mass and support fatigue recovery, demonstrated the highest abundance in the river group. This compound could support the intense exercise of *E. sinensis* in the river group [50]. We also identified several metabolites associated with flavor, such as ethyl 4-methylphenoxyacetate and ganoderic acid. These metabolites might have contributed to the special taste of *E. sinensis* collected from the Yangtze River [46]. In the lake group, the abundance of unsaturated fatty acids, such as DHA, 15(S)-hydroxyeicosatrienoic acid, and 13(S)-HpODE, and intermediates of amino acid metabolism, was the highest. The previous study highlighted the concentrations of the total free amino acids, umami 5′-nucleotide compounds, and polyunsaturated fatty acids in the hepatopancreas and muscles of pond-produced crabs were significantly lower than those of lake-produced ones [45]. This result might be influenced by the good water quality, balanced diet resources and abundance, and appropriate water temperature of Yangcheng Lake [41]. In the pond group, riboflavin, nobiletin, and geniposidic acid displayed the highest abundance, and these metabolites were positively correlated with Cyanobacteria in the gut microbiome (Fig. S8). The high abundance of Cyanobacteria was associated with the eutrophic conditions in the aquaculture pond [43]. In sum, three diverse muscle metabolomes with differential metabolites were identified, which indicated the high plasticity of the muscle metabolome of *E. sinensis* in the pond, lake, and river environments.

The correlation analysis indicated that the abundance of gut microbiome communities was correlated with the abundance of metabolites in muscle metabolome. This result implies that the gut microbiome and muscle metabolome were not independently shaped by the environment. In fact, they were related and interacted with each other. This inference reflected the high plasticity of *E. sinensis*. The high phenotypic plasticity of *E. sinensis* could contribute to the acclimation and adaptation to different and even introduced environments.

## Supplemental Materials



Supplementary data for this paper are available on-line only at http://jmb.or.kr.
