# Supplementary material for: High Plasticity of the Gut Microbiome and Muscle Metabolome of Chinese Mitten Crab (Eriocheir sinensis) in Diverse Environments
Source: J Microbiol Biotechnol. 2020 Nov 16;31(2):240–9. doi: 10.4014/jmb.2011.11018 (PMC9705879; doi:10.4014/jmb.2011.11018)
Supplement: Supplementary file 1 [file jmb-31-2-240-supple.pdf]

J. Microbiol. Biotechnol.
https://doi.org/10.4014/jmb.2011.11018

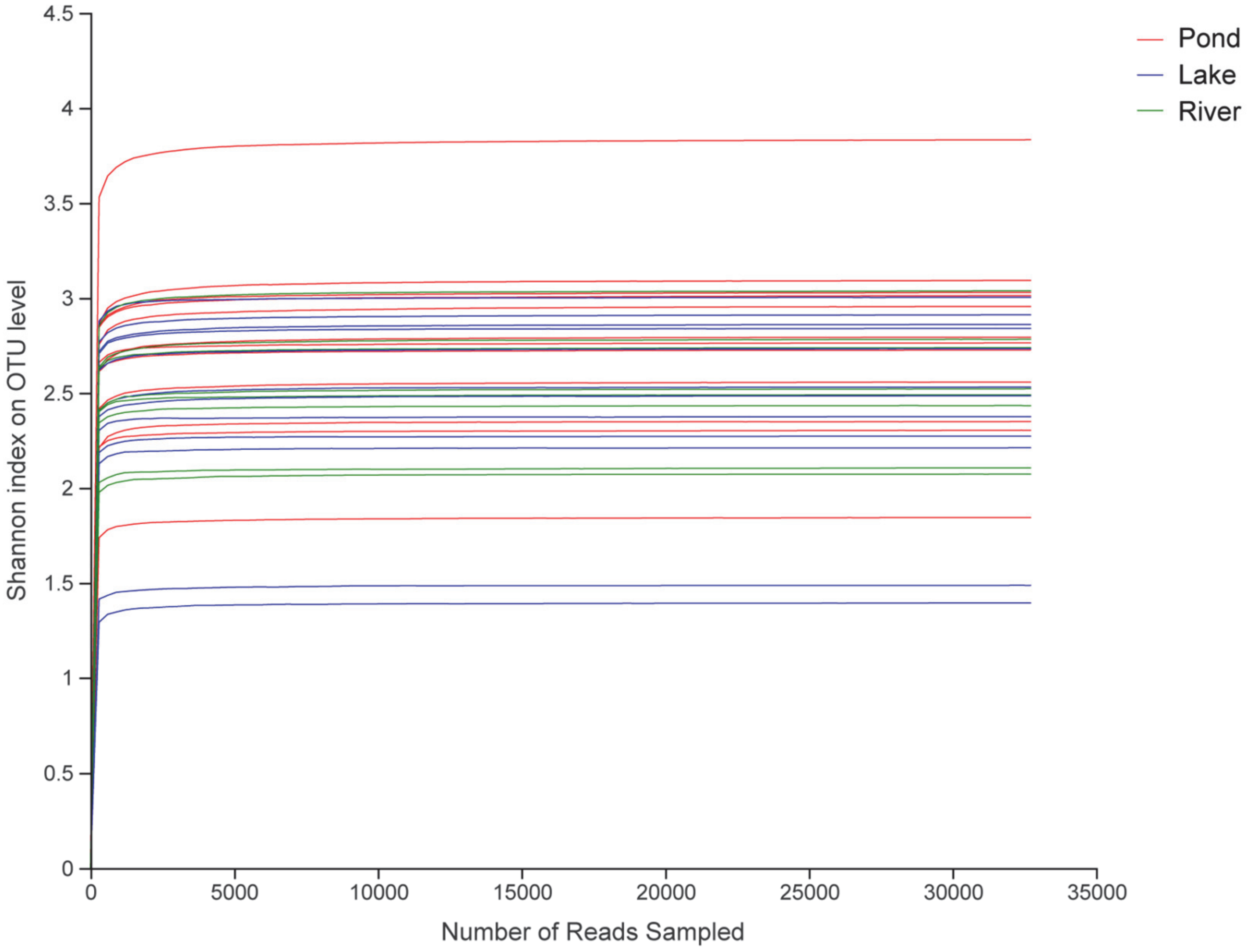

Figure S1. Shannon rarefaction curve of the read numbers and Shannon indices at the OTU level.

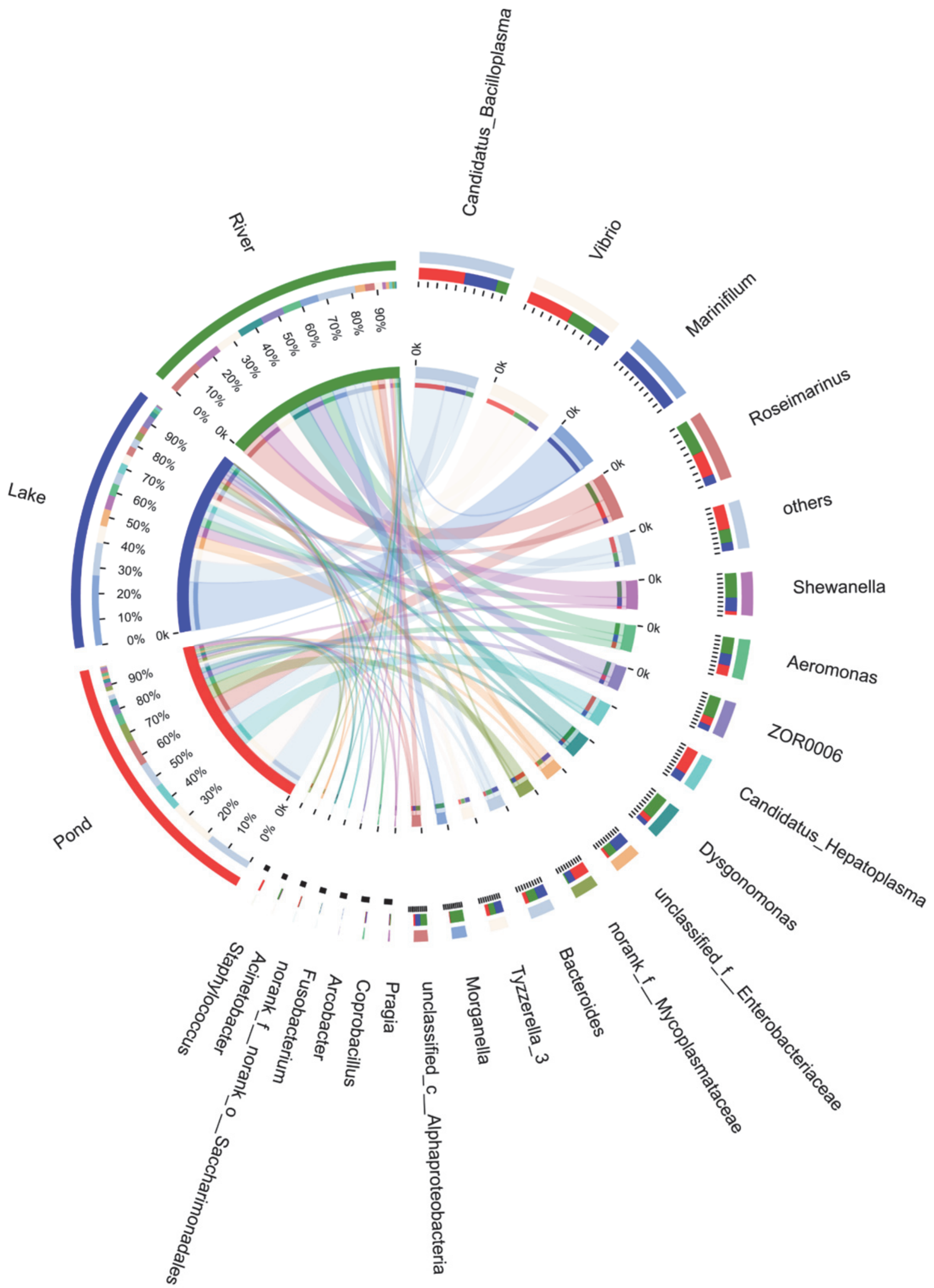

Figure S2. Circos plot of the proportion of gut microbiome in the three environmental groups at genus level.

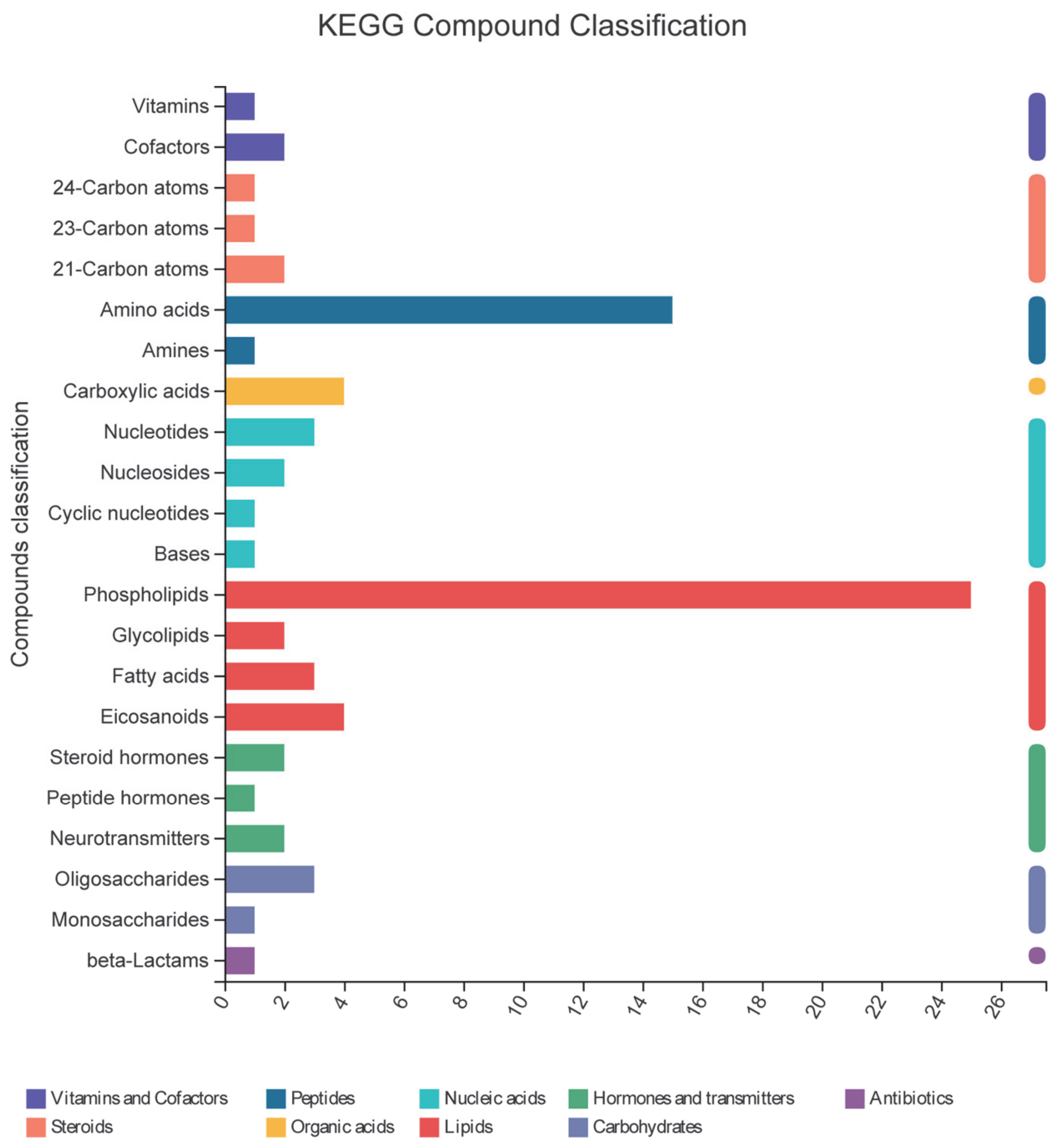

Figure S3. KEGG compound classification for the total identified metabolites.

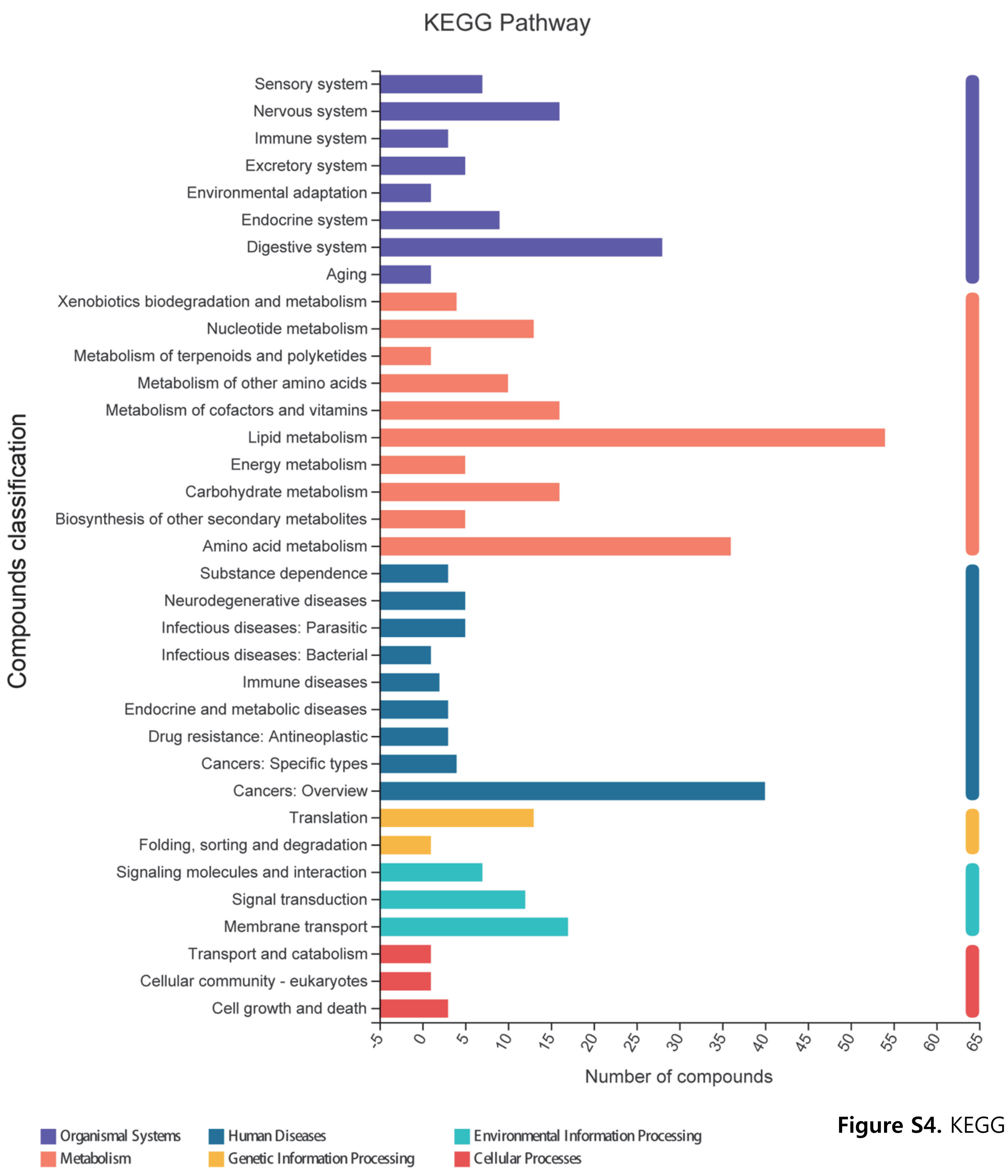

Figure S4. KEGG pathway annotation for the total identified metabolites.

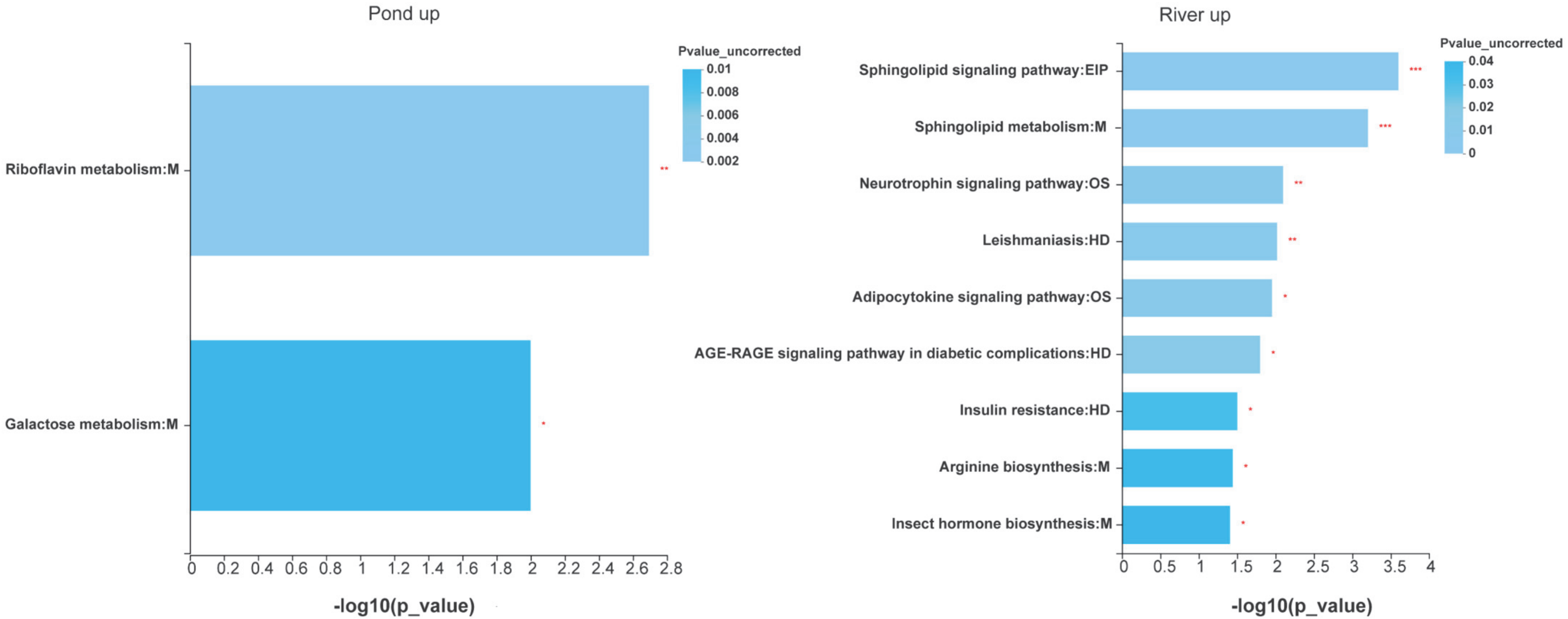

Figure S5. KEGG enrichment analysis for differential metabolites identified between the pond and river groups.Note:* $P < 0.05$, ** $P < 0.01$,*** $P < 0.001$.

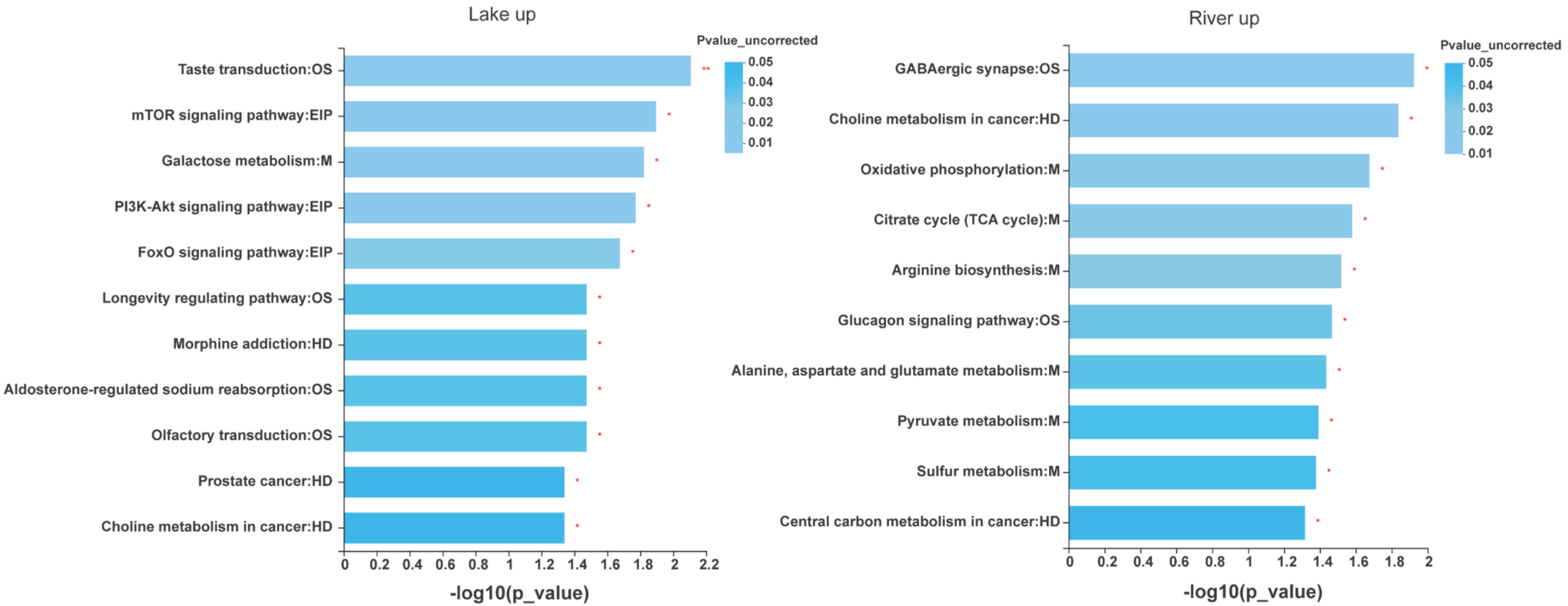

Figure S6. KEGG enrichment analysis for differential metabolites identified between the lake and river groups.Note:* $P < 0.05$, ** $P < 0.01$,*** $P < 0.001$.

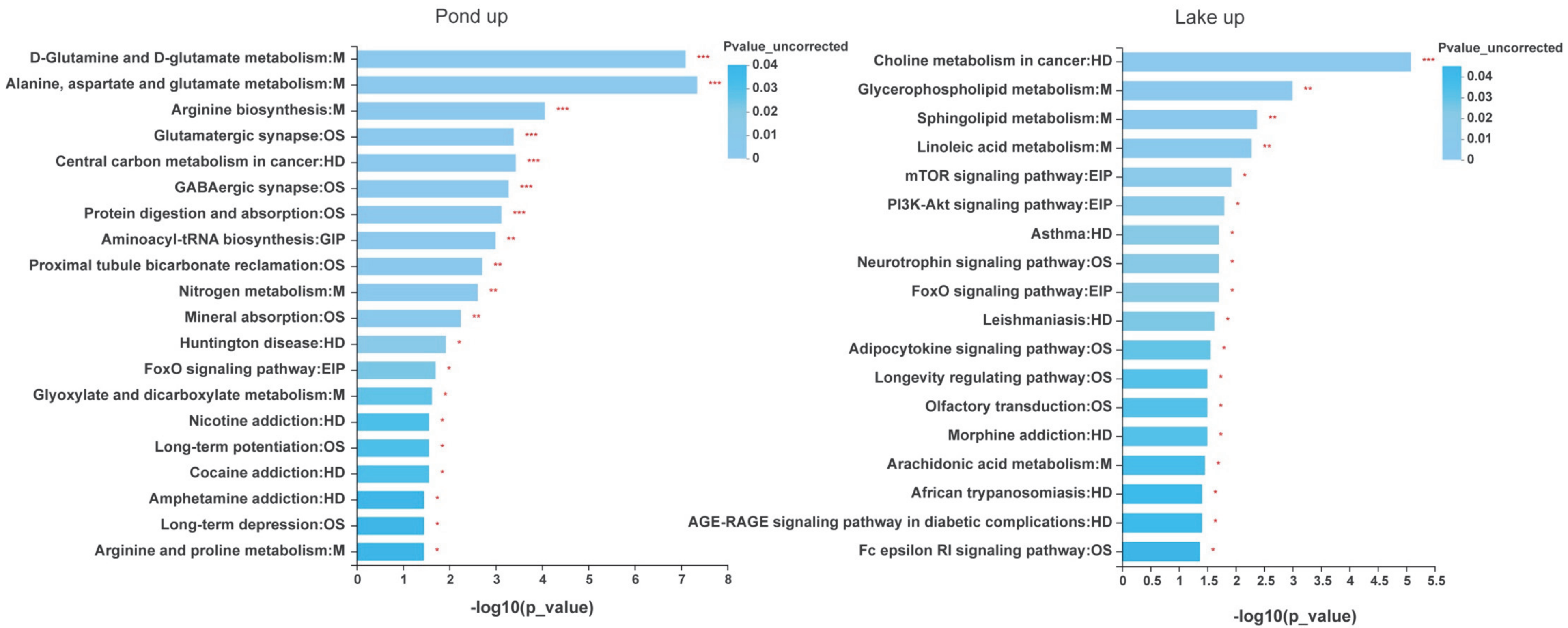

Figure S7. KEGG enrichment analysis for differential metabolites identified between the pond and lake groups.Note:* $P < 0.05$, ** $P < 0.01$,*** $P < 0.001$.

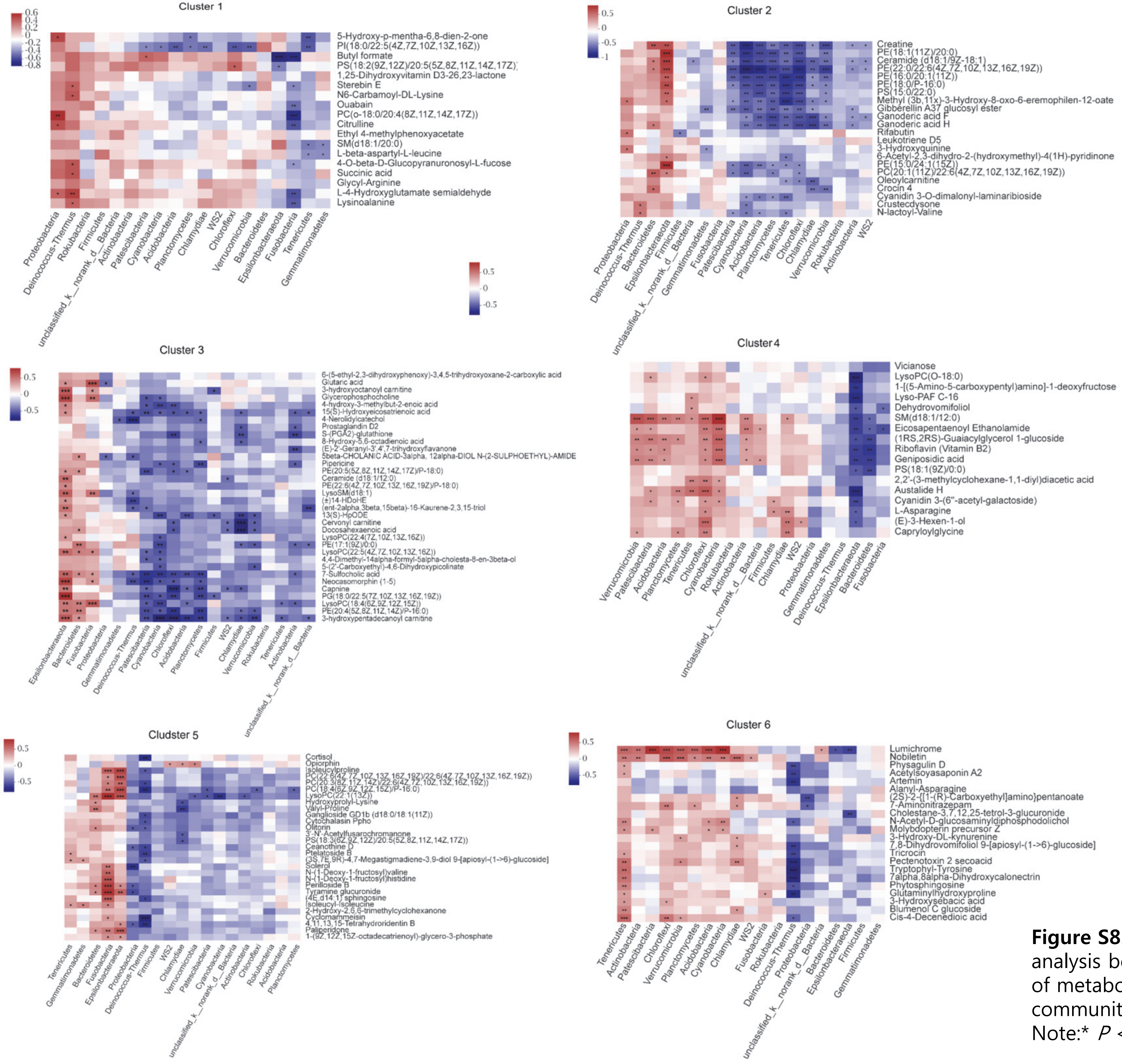

Figure S8. Spearman's correlation analysis between the abundance of metabolites and gut microbiome communities in the identified clusters. Note:* $P < 0.05$, ** $P < 0.01$,*** $P < 0.001$.
